# Supplementary material for: Ultra-broadband optical amplification using nonlinear integrated waveguides
Source: Nature. 2025 Apr 9;640(8060):918–23. doi: 10.1038/s41586-025-08824-3 (PMC12018452; doi:10.1038/s41586-025-08824-3)
Supplement: Supplementary file 1 — Supplementary Information [file 41586_2025_8824_MOESM1_ESM.pdf]

nature portfolio

https://doi.org/10.1038/s41586-025-08824-3

Supplementary information

Ultra-broadband optical amplification using nonlinear integrated waveguides

In the format provided by the authors and unedited

Supplementary Information

Ultra-broadband optical amplification using nonlinear integrated waveguides

Ping Zhao[1,2], Vijay Shekhawat[1], Marcello Girardi[1], Zonglong He[1], Victor Torres-Company[1], and Peter A. Andrekson[1]

[1]Photonics Laboratory, Department of Microtechnology and Nanoscience, Chalmers University of Technology Gothenburg 41296, Sweden.

[2]College of Electronics and Information Engineering, Sichuan University, Chengdu 610065, China.

I. Balance between loss, nonlinearity and dispersion for parametric process

For the Si_3N_4 nonlinear platform, we find that anomalous dispersion is obtained mainly when the total thickness of the rib waveguide is more than 650 nm through typical simulations. Generally, the maximum thickness of crack-less Si_3N_4 thin film we can achieve is about 800 nm via direct LPCVD on a flat silica substrate. Here, we focus on 800-nm-thick Si_3N_4 wafer. Although the confinement of the modes for this thickness may not be the highest, one advantage of maximizing the achievable waveguide thickness is that the bending technique of cutting off high-order modes in the rib Si_3N_4 waveguides would perform better for a moderate confinement. Figure S1 presents the simulated nonlinear coefficient (blue) of TE_{00} mode and required width (red) of a straight rib Si_3N_4 waveguide varying with the slab thickness ratio at a targeted zero-dispersion wavelength of 1550 nm. The total thickness of the rib Si_3N_4 waveguide, i.e., H_1+H_2, is 800 nm. As can be seen in Fig. S1, the required rib width increases with slab thickness. Besides, the nonlinear coefficient drops when the slab thickness ratio increases, which is attributed to the field confinement decrease. The propagation loss (α) of the rib Si_3N_4 waveguide we fabricated is mainly determined by the rib sidewall roughness as well as the overlap area between optical field and the sidewall[1,2], if the nanoparticle issue is fully solved. A higher slab thickness ratio would lead to a lower propagation loss which corresponds to a larger effective nonlinear length, $L_{eff} = (1-e^{-\alpha L})/\alpha$, where L is the physical waveguide length[3]. At phase matching wavelengths, the gain of a waveguide parametric amplifier is proportional to the exponential term[4], $e^{-\alpha L+2P\gamma L_{eff}}$. As a result, a trade-off between nonlinearity and loss needs to be taken. We picked a slab ratio of 62.5%, i.e., H_1 = 300 nm and H_2 = 500 nm, for a strategy that the nonlinear coefficient is still large, and the propagation loss would be small. Other slab ratios would be explored in our future work.

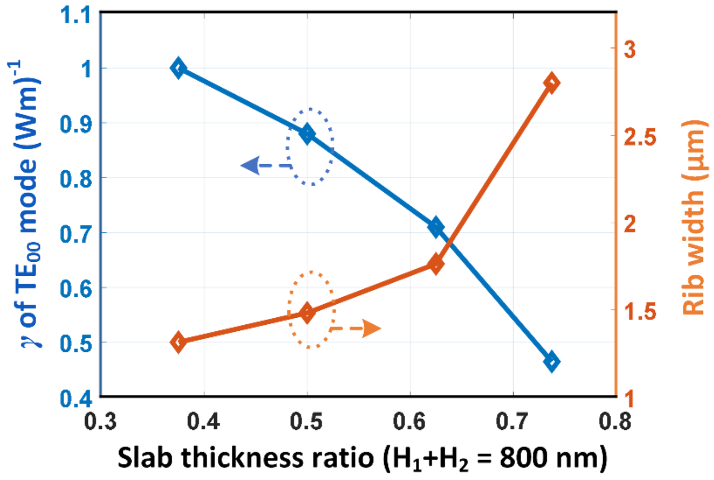

Figure S1. Nonlinear coefficient of TE_{00} mode (blue line) and required rib width (red line) varying with slab thickness ratio for a total rib Si_3N_4 waveguide thickness of 800 nm when the targeted zero-dispersion wavelength is 1550 nm.

II. Maintaining single mode in rib waveguides with finite slab width

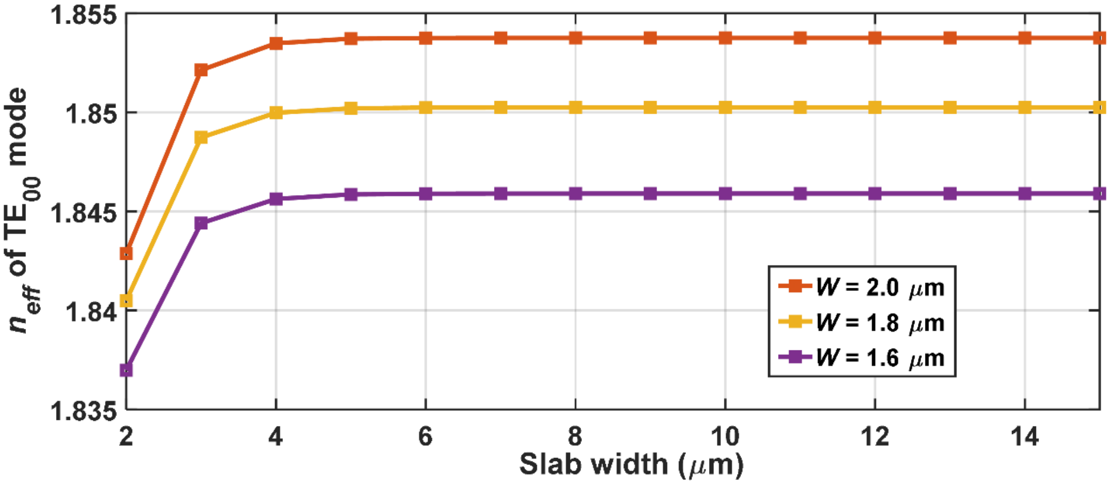

Figure S2. Simulated effective refractive index of TE_{00} mode varying with the slab width of different straight rib Si_3N_4 nonlinear integrated waveguides at 1550 nm. The purple, yellow and red curves correspond to rib widths of 1.6 μm, 1.8 μm and 2.0 μm, respectively.

Ideally, a bent rib Si_3N_4 waveguide can be single mode if the slab is infinitely wide. When its thickness is more than 400 nm, practical Si_3N_4 slab is likely to crack due to huge temperature change during fabrication. In this work, we etched a part of the slab to release the stress of Si_3N_4 and obtained a groove in the slab layer. As a result, the slab width is finite, and slab modes are supported in the fabricated rib Si_3N_4 nonlinear integrated waveguides. The coupling between the fundamental and slab modes can be efficiently avoided by giving a wide Si_3N_4 slab. Figure S2 shows the simulated effective refractive index of TE_{00} mode as a function of the slab width of straight rib Si_3N_4 nonlinear integrated waveguides. The thicknesses of the rib and slab are 300 nm and 500 nm. The purple, yellow and red lines correspond to the rib width of 1.6 μm, 1.8 μm and 2.0 μm, respectively. As can be seen in Fig. S2, the TE_{00} effective refractive index is constant when the slab width is larger than 6 μm. This indicates that the TE_{00} mode will not be disturbed by the slab with a sufficiently large width. We optimized the nanofabrication processes and obtained smooth rib Si_3N_4 waveguides with slab widths of more than 12 μm, considering different bent radii. In this manner, single-mode transmission was achieved in the sub-meter-long rib Si_3N_4 nonlinear integrated waveguides. Besides, the spacing between adjacent rib spiral waveguides equals the slab width plus the width of the groove in the slab and is sufficiently large so that coupling in each spiral unit is avoided.

III. Dispersion in a spiral rib waveguide

The local radius of the proposed single-mode nonlinear waveguide changes along the waveguide. Figure S3 shows the simulated 2nd-order dispersion of TE_{00} mode varying with the radius of a rib Si_3N_4 nonlinear integrated waveguide at 1550 nm wavelength. The dimension parameters of the rib Si_3N_4 waveguide cross section are W = 1.9 μm, H_1 = 300 nm and H_2 = 500 nm. For the 56-cm-long single-mode rib Si_3N_4 nonlinear integrated waveguide used in the experiment, the radius of the 80% waveguide in one spiral unit is in the shallow-yellow regime. As can be seen in Fig. S3, the designed second- and fourth-order dispersion is about -4 ps^2/km and 1.75 fs^4/ μm, respectively, which is in good agreement with the FWM measurements. Besides, the second-order dispersion is more sensitive to the bent radius of the rib Si_3N_4 nonlinear integrated waveguide than the fourth-order dispersion. We tried to use large bent radius to reduce the dispersion variation along the waveguide. Currently, the maximum radius is limited by the EBL writing field, i.e., 1 mm x 1 mm.

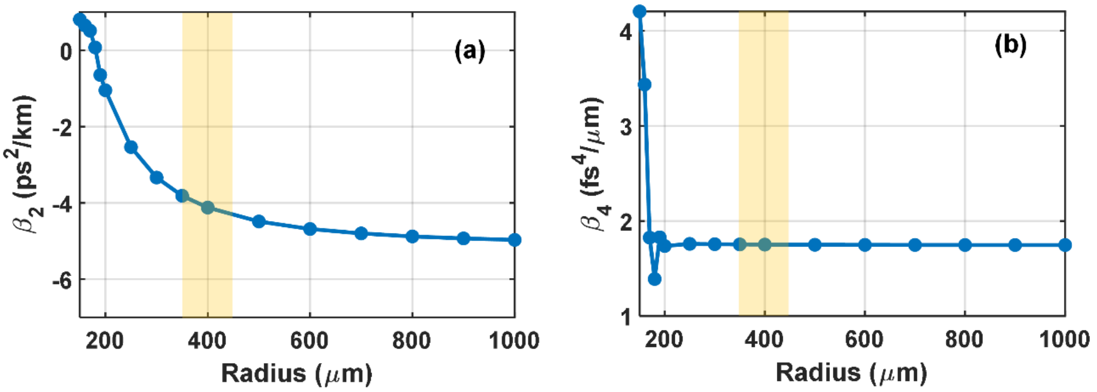

Figure S3. (a) Second- and (b) fourth-order dispersion as a function of the radius of a rib Si_3N_4 nonlinear integrated waveguide at 1550 nm for TE_{00} mode. The waveguide parameters of the cross section are W = 1.9 μm, H_1 = 300 nm and H_2 = 500 nm. The shallow-yellow regime corresponds to the radius range of eighty percent of the waveguide.

IV. Ultra-wideband PF spectra

Although we used three widely wavelength-tunable ECLs (1355 nm ~ 1495 nm, 1480 nm ~ 1640 nm and 1580 nm ~ 1680 nm) for the FWM characterization, the measurement of the full parametric bandwidth of the 56-cm-long single-mode rib Si_3N_4 nonlinear integrated waveguide can still not be made due to limited single laser wavelength range. An alternative method to estimate the parametric bandwidth is to check the PF spectrum of the Si_3N_4 nonlinear integrated waveguide. By only feeding the continuous-wave pump wave into the Si_3N_4 nonlinear integrated waveguide, we achieved pure PF due to spontaneous FWM emission in the Si_3N_4 nonlinear waveguide. Figure S4 shows the PF spectra with pump wavelengths of 1544.4 nm (blue), 1549.1 nm (red) and 1551.6 nm (yellow). The on-chip pump power was about 34 dBm in TE_{00} mode. The two PF peaks at the spectrum edges correspond to phase match wavelengths due to fourth-order dispersion for the 1544.4 nm pump wavelength. As is shown in Fig. S4, the PF flatness improves when the pump wavelength increases. The second- and fourth-order dispersion changes with the pump wavelength, which can be seen in Fig. E1 (a) and (b). Hence, there is an optimum pump wavelength to obtain a spectrally flat PF. The improvement of PF flatness is in agreement with theoretical expectations shown by Fig. 2(a). The PF flatness was also affected by the integrated tapers which were mainly optimized in the C and L bands.

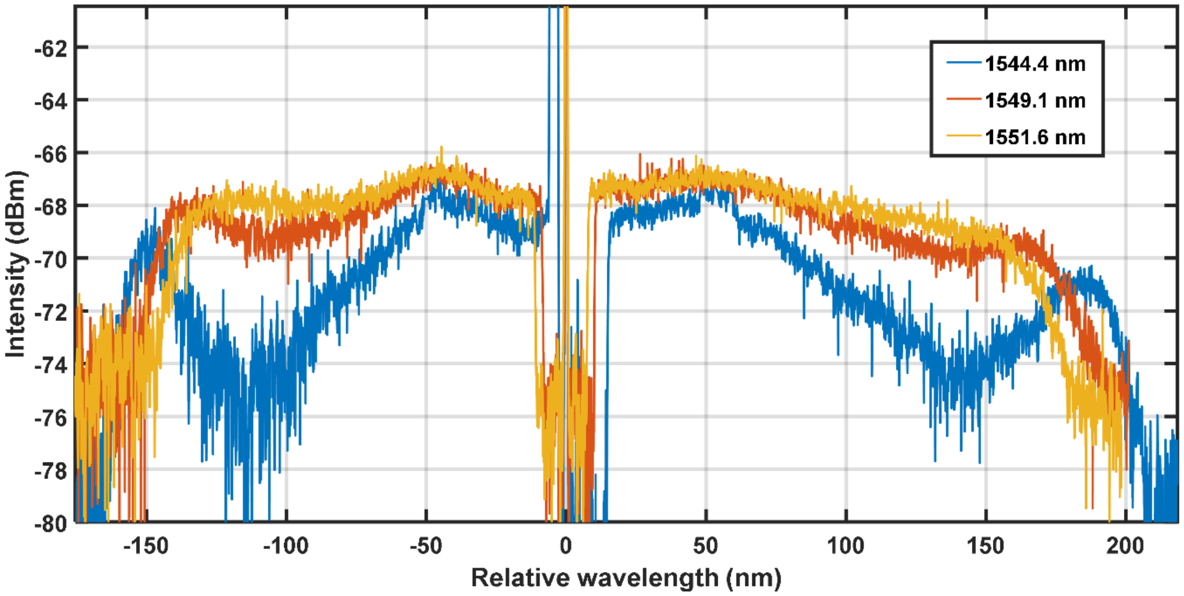

Figure S4. Measured PF spectra with pump wavelengths of 1544.4 nm (blue), 1549.1 nm (red) and 1551.6 nm (yellow). The resolution of optical spectrum analyzer was 0.2 nm. The on-chip pump power was 34 dBm.

V. Fabrication tolerance to OPA spectra

Figure S5 (a) and (b) show a theoretical analysis of tolerance on the rib width and height for a silicon nitride waveguide OPA, respectively. The optimized gain spectrum is the red solid curve with a rib width of 1828 nm and a rib thickness of 300 nm. The waveguide loss is 0.6 dB/m and the length of the waveguide is 2 m with a pump power of 34 dBm. We consider a typical width uncertainty of +/- 5 nm for EBL. When the width increases from 1823 nm to 1833 nm, the corresponding bandwidth as well as the gain flatness changes, as can be seen in Fig. S5 (a). When the rib width is 1780 nm, the second-order dispersion becomes normal (β_2 > 0). In this case, phase matching is not satisfied and results in a low gain/CE and a narrow bandwidth. The OPA spectrum is more sensitive to the height variation, as can be seen in Fig. S5 (b), which is due to that the waveguide has stronger field confinement in height direction. Wafer planarization techniques, such as chemical mechanical polishing which can lead to a surface roughness down to 0.2 nm[5], would be useful to fabricate wideband high-gain waveguide OPAs.

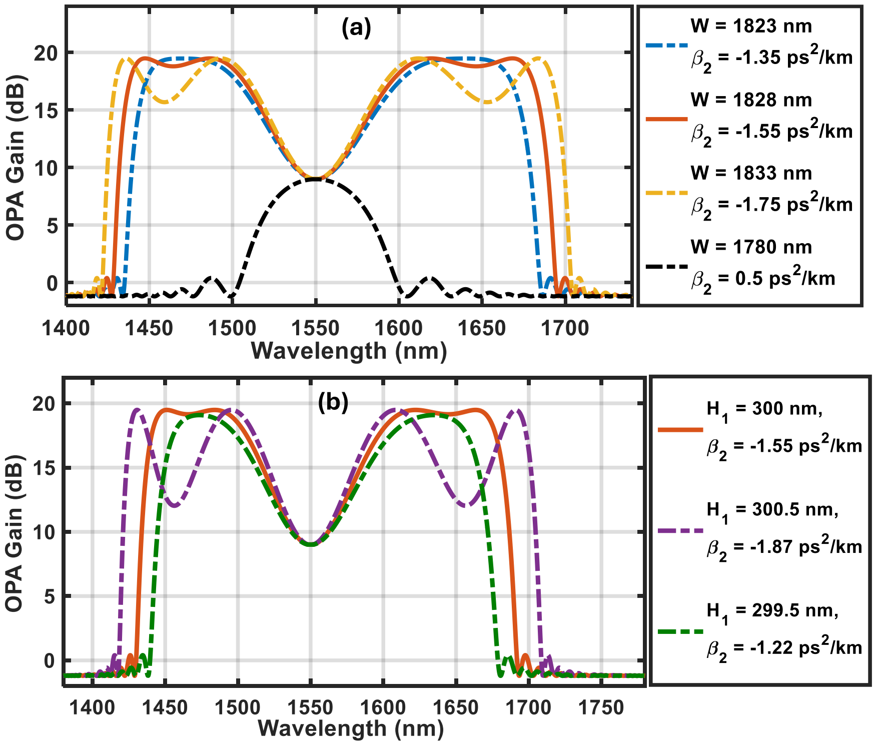

Figure S5. Theoretical gain spectra of single-mode silicon nitride rib waveguide OPAs with fabrication uncertainties in waveguide (a) width and (b) height. The slab thickness is 500 nm with a CW 1550 nm pump power of 34 dBm. The waveguide loss is 0.6 dB/m and the length of the waveguide is 2 m.

VI. Field propagation through a bend waveguide

Figure S6 shows simulated intensity profiles of the electrical field of a 1550-nm optical wave transmitting along different rib waveguides with various eigen modes excited at the input port. The shared waveguide geometry parameters are W = 1.9 μm, H_1 = 300 nm and H_2 = 500 nm. A FDTD solver from Ansys Lumerical was utilized for the simulation. Figure S6 (a) and (b) are for a straight waveguide, from which we can see that the transmission of both TE_{00} and TE_{10} modes is lossless. For the rib waveguide used in Fig. S6 (c) and (d), it was about 600 μm long and the bending radii at the start and end ports are infinite and 400 μm, respectively. The bend rib waveguide was adiabatically designed for TE_{00} mode. As can be seen in Fig. S6 (c) and (d), the transmission of the TE_{00} mode is 100% while the TE_{10} mode is almost attenuated after the propagation along such a bend waveguide.

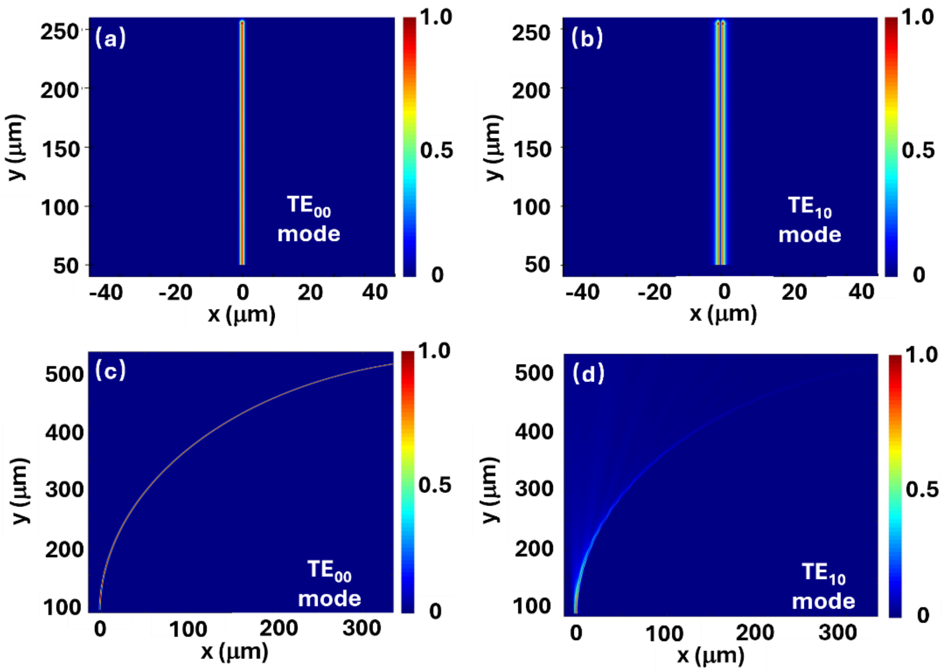

Figure S6. Simulated intensity profile of the electrical field of a 1550-nm optical wave propagating along straight (a, b) and (c, d) rib waveguides with TE_{00} (a, c) and TE_{10} (b, d) eigen modes excited at the waveguide input, respectively. The simulation tool was based on a FDTD solver.

Reference

1 Payne, F. P. & Lacey, J. P. R. A theoretical analysis of scattering loss from planar optical waveguides. *Opt Quantum Electron* **26**, 977 (1994).

2 Lee, K. K., Lim, D. R., Luan, H.-C., Agarwal, A., Foresi, J., & Kimerling, L. C. Effect of size and roughness on light transmission in a Si/SiO_2 waveguide: Experiments and model. *Appl Phys Lett* **77**, 1617–1619 (2000).

3 Agrawal, G. *Nonlinear Fiber Optics*, (Elsevier, 2009).

4 Karlsson, M., Schröder, J., Zhao, P., & Andrekson, P. A. in *Conference on Lasers and Electro-Optics (CLEO) 2021*, p. JTh3A.5.

5 Ji, X., et al. Ultra-low-loss on-chip resonators with sub-milliwatt parametric oscillation threshold. *Optica* **4**, 619-624 (2017).
